# A case of acquired angioedema associated with Waldenstrom’s macroglobulinemia treated with rituximab

**DOI:** 10.1186/1710-1492-10-S2-A41

**Published:** 2014-12-18

**Authors:** Caroline Rizk, William H Yang, Jason Tay, Jacob Karsh

**Affiliations:** 1 Department of Clinical Immunology and Allergy, McGill University, Montreal, QC Canada; 2 Department of Internal Medicine, University of Ottawa, Ottawa, ON, Canada; 3 Ottawa Allergy Research Corporation, Ottawa, ON, Canada

## Background

Acquired angioedema (AAE) is a rare disorder caused by acquired deficiency of C1 inhibitor (C1-INH), very often seen in association with lymphoproliferative diseases. The key elements of AAE are acquired deficiency of C1 inhibitor, hyperactivation of the classical pathway of complement, and recurrent angioedema symptoms.

## Case presentation

We report a case of Waldenstrom´s macroglobulinemia causing an acquired deficiency of C1 esterase inhibitor in a 40-year-old woman. She initially presented with an episode of angioedema followed by many episodes of abdominal distention associated with pain, vomiting, and diarrhea for 1.5 years. Work-up revealed low C1 esterase inhibitor levels, normal C3, and nonexistent C4. A diagnosis of acquired C1 esterase inhibitor deficiency was proposed at the time. However, it was also noted that her IgM was very elevated with an IgM monoclonal gammopathy with kappa light chains, and an enlarged spleen. Bone marrow biopsy and aspirate revealed clonal B-cells staining positively for CD20. She was diagnosed with Waldenstrom´s macroglobulinemia in association with C1 esterase inhibitor deficiency. Although the association is recognized, it is rare, and likely secondary to antibodies against C1 esterase inhibitor. After treatment with rituximab, cyclophosphamide, and prednisone, her paraprotein levels fell to normal range, and her autoimmune parameters normalized with significant clinical improvement. She has not had any further episodes of angioedema. She will continue on rituximab as maintenance therapy for 2 more years.

## Conclusion

Acquired deficiency of C1 esterase inhibitor is quite rare with just over 100 cases in the literature. Given that most cases are related to antibodies against C1 esterase inhibitor, rituximab may be the treatment of choice. However, treatment of the underlying lymphoproliferative disorder may need to be considered as well and could provide a more definitive treatment.

## Consent

Written informed consent was obtained from the patient for publication of this abstract and any accompanying images. A copy of the written consent is available for review by the Editor of this journal.

